# Evaluation of cytokine levels in HIV-infected individuals on therapy with tenofovir, lamivudine, and dolutegravir

**DOI:** 10.1590/1414-431X2025e14442

**Published:** 2025-05-09

**Authors:** C.D. da Silva-Junior, B.A. Silva, J.P. Gonçales, M.M. da Silva, L.R. Moreira, M.S. Barros, M.C.S. Rabello, P.S.R. de Araújo, V.M.B. de Lorena, L.C.R.V. Moura

**Affiliations:** 1 Departamento de Medicina Tropical, Universidade Federal do Pernambuco, Recife, PE, Brasil; 2 Instituto Aggeu Magalhães, Fundação Oswaldo Cruz - FIOCRUZ, Recife, PE, Brasil; 3 Grupo SER Educacional, Recife, PE, Brasil; 4 Hospital Correia Picanço, Recife, PE, Brasil

**Keywords:** Antiretroviral therapy, CD4^+^ lymphocytes, Inflammatory response, Cytokine modulation

## Abstract

Antiretroviral therapy (ART) is essential to reduce viral load and restore CD4^+^ T cell levels in people living with HIV/AIDS (PLWHA). However, different treatment protocols influence the levels of cytokines, important mediators of the immune response. This study aimed to evaluate cytokine levels in PLWHA on therapy with tenofovir (TDF), lamivudine (3TC), and dolutegravir (DTG). The results showed that PLWHA on treatment had a significant increase in CD4^+^ T lymphocyte levels and a reduction in CD8^+^ T lymphocyte levels compared to naive (untreated) individuals. Furthermore, PLWHA treated with TDF/3TC/DTG had a significant reduction in interleukin (IL)-4 and IL-10 levels (P<0.02; P=0.047) compared to other ART regimens. Naive individuals had higher levels of IL-2 and interferon (IFN)-γ, while their levels of tumor necrosis factor (TNF), IL-4, and IL-10 were lower. These findings suggested that TDF/3TC/DTG treatment modulated cytokines, reducing chronic inflammation and improving the immune response in PLWHA. The decrease in anti-inflammatory cytokines, such as IL-4 and IL-10, may be associated with better regulation of the immune system, resulting in greater control of infection and a balanced inflammatory response.

## Introduction

Four decades after the first cases were described, human immunodeficiency virus (HIV) infection is still one of the significant public health problems worldwide due to its pandemic nature and severity (1). The leading cause of morbidity and mortality of people living with HIV/AIDS (PLWHA) is associated with acquired immunodeficiency syndrome (AIDS). This phase is characterized by a progressive decrease in CD4^+^ T lymphocyte levels and an increase in viral load in the bloodstream and lymphoid organs, triggering several alterations in the immune system and consequently the occurrence of opportunistic infections characteristic of AIDS (2).

Combined antiretroviral treatment (c-ART) consists of using a combination of three different classes of drugs to reduce viral load and restore immune functions, even if only partially (3). Under c-ART, a decrease in mortality from opportunistic diseases has been observed, but there is a proportional increase in non-AIDS diseases such as cardiovascular disease, liver disease, and kidney disease. In Brazil, the preferred c-ART regimen is based on the combination of two nucleotide analog reverse transcriptase inhibitors (NRTI): tenofovir (TDF) and lamivudine (3TC) and an integrase inhibitor (INI): dolutegravir (DTG) (4).

Studies have shown that in addition to significantly inhibiting viral replication in PLWHIV, c-ART can alter the immune system's functionality, especially the expression of cytokines. Akase et al. (5) evaluated the effects of ART and observed high levels of interleukin (IL)-6 and IL-10 compared to ART naive with ART experienced. In 2018, a study by Osuji et al. (6) evaluated the effects of combined therapy with TDF/3TC and efavirenz (EFZ) and observed that after 12 months of treatment, there were reductions in tumor necrosis factor (TNF), IL-4, IL- 6, IL-10, and transforming growth factor-beta (TGF-β) and an increase in interferon-gamma (IFN-γ) levels. However, a study by Bordoni et al. (7) observed that, after c-ART that included several classes of drugs, levels of IL-2, IL-5, IL-7, IL-9, and IL-18 decreased, but no effect on IL-6, IL-10, and IL-13 was demonstrated. In addition, Szymańska et al. (8) observed that using 2 NRTI combined with protease inhibitors (PI) or INI decreased IL-4, IL-7, and IL-15, with no difference compared to PI and INI.

In this scenario, we observed studies that evaluated the role of cytokines in persistent inflammation and immune activation, described as predictors of clinical events and mortality in HIV-infected patients. Additionally, available data on the impact of c-ART on cytokine levels are still controversial, mainly due to the availability of different combinations and classes of drugs that may play different roles in inflammation and immune activation. Therefore, this study aimed to evaluate the impact of the TDF/3TC/DTG regimen by measuring inflammatory and anti-inflammatory cytokines in serum samples from PLWHA.

## Material and Methods

### Study population

Peripheral blood samples from 87 PLWHA were collected at the Clinical Hospital of the Federal University of Pernambuco (Recife, Brazil) and at the Correia Picanço Hospital (HCP; Recife, Brazil), from November 2019 to February 2020. The individuals were of both genders and aged between 18 and 65 years and were included in the study according to the following criteria: i) had started antiretroviral treatment after February 2017 and had been on treatment for six months; ii) had an undetectable viral load; iii) CD4^+^ T lymphocyte values >200 cells/mm^3^; iv) had never changed treatment regimen. Exclusion criteria were: i) having a clinical and laboratory diagnosis for syphilis, hepatitis B and C, or tuberculosis; ii) diabetes and other immune-mediated diseases; iii) pregnant.

The treatment regimen was used to categorize the study groups: DTG group (n=42) that received the tenofovir, lamivudine, and dolutegravir regimen and TARV group (n=25) that received other treatment regimens, which did not include dolutegravir. In addition, we also evaluated a group of 20 individuals (NAIVE group) with a confirmed diagnosis of HIV in a health service center and referred to the HCP, but needed to repeat the test using two subsequent rapid tests before starting treatment: ABON^TM^ HIV 1/2/0 Tri-line rapid test (Abott Diagnósticos Rápidos S.A, Brazil) and HIV 1/2 Bio-Manguinhos^®^ (Brazil) rapid test, with Dual Path Platform (DPP^®^) technology (Bio-Manguinhos/FIOCRUZ) available in the hospital's clinical analysis laboratory. After confirmation, the individuals were invited to participate in the study, a peripheral blood sample was collected, and we followed the same exclusion criteria as the other patients, in addition to presenting CD4^+^ T lymphocyte levels >200 cells/mm^3^ (Table 1).

**Table 1:** Characteristics of the analyzed groups.

	NAIVE	DTG	TARV	P	
n	20	42	25		
Age (years)	30 (27-36.7)	38 (33-43,2)	37 (28.5-47)	**NAIVE vs DTG**	**0.002**a
				**NAIVE vs TARV**	**0.020**a
				DTG *vs* TARV	0.972^a^
Gender	M=18	M=36	M=13	-	
	F=2	F=10	F=12		
Treatment	-	TDF + 3TC + DTG	TDF/3TC/EFV (n=10)	-	
			TDF/3TC/RIT/ATA (n=5)		
			3TC/RAL/ABC (n=6)		
			AZT/3TC/ATA/RIT (n=4)		
Viral load (copies/mL)	44863 (8732-181313)	n.d.	n.d.	-	
CD4^+^ T Cells (cells/mm^3^)	424 (303.3-535.8)	587 (362-812.8)	566 (394-729.5)	**NAIVE vs DTG**	**0.009a**
				NAIVE *vs* TARV	0.062^a^
				DTG *vs* TARV	0.578^a^
CD8^+^ T Cells (cells/mm^3^)	1529 (970.8-1718)	896 (700-1040)	764 (542- 974)	**NAIVE vs DTG**	**0.0003a**
				**NAIVE vs TARV**	**<0.0001**
				DTG *vs* TARV	0.079^a^
CD4:CD8	0.31 (0.17-0.50)	0.76 (0.47-1.00)	0.665 (0.49-1.32)	**NAIVE vs DTG**	**<0.0001b**
				**NAIVE vs TARV**	**<0.0001b**
				DTG *vs* TARV	0.608^b^
Duration of treatment (weeks)	-	77.5 (55-112.8)	106 (69.5-117)	NAIVE *vs* DTG	-
				NAIVE *vs* TARV	-
				DTG *vs* TARV	0.175^a^

Data are reported as median and interquartile range. ^a^Unpaired *t*-test with Welch's correction and ^b^Mann Whitney test. Data in bold type are statistically significant (P<0.05). M: male; F: female; n.d.: undetectable; TDF: tenofovir; 3TC: lamivudine; DTG: dolutegravir; EFV: efavirenz; RIT: ritonavir; RAL: raltegravir; ABC: abacavir; AZT: zidovudine; ATA: atazanavir.

### Blood collection and sample processing

Peripheral blood (10 mL) was collected from patients using a vacuum system (Vacutainer^®^, USA) into tubes without anticoagulants. The samples were centrifuged at 250 *g* for 5 min at 21°C to obtain the serum. Then, the serum was aliquoted in a volume of 500 μL placed in microtubes and frozen at -80°C until Cytometric Bead Array (CBA) analysis. The samples were processed and stored in the Immunoparasitology Laboratory Aggeu Magalhães Institute, FIOCRUZ (Brazil).

### Determination of serum cytokine concentration

The concentrations of cytokines (IFNγ, TNF, IL-2, IL-4, IL-6, and IL-10) were measured using the BD™ CBA human Th1/Th2 cytokine kit II (USA) according to the manufacturer's recommendations. Samples were acquired with a FACSCalibur™ flow cytometer (Becton Dickson Immunocytometry Systems, USA) located at the Nucleus of Technological Platforms (NPT)/IAM/Fiocruz. Approximately 300 events were acquired per capture bead. Analysis was performed using FCAP Array™ 3.1 software (Beckton Dickson), and results are reported in picograms per milliliter (pg/mL).

### Statistical analysis

The continuous variables were presented as medians and interquartile ranges (IQR). The levels of TNF, IFN-γ, IL-2, IL-4, IL-6, and IL-10 showed a non-normal distribution according to the Shapiro-Wilk test. Consequently, the Mann-Whitney nonparametric test was used for comparisons between two groups. Spearman's correlation test was applied to analyze relationships, given the non-normal distribution of data. Correlations were categorized based on the r value: negligible (≥0.00 to ≤0.30), low (>0.30 to ≤0.50), moderate (>0.50 to ≤0.70), high (>0.70 to ≤0.90), and extremely high (>0.90 to ≤1.00). Samples with undetectable values were excluded from the analysis. Data analysis was conducted using GraphPad Prism version 10.0, with significance set at P<0.05.

### Ethical considerations

All included patients signed the informed consent form, and the approaches used in this study were approved by the Research Ethics Committee of the IAM/FIOCRUZ (protocol number CAAE: 12152019.4.3001.8807).

## Results

In the present study, the DTG group exhibited higher levels of CD4^+^ T lymphocytes compared to the NAIVE group. However, the NAIVE group demonstrated elevated levels of CD8^+^ T lymphocytes and a higher CD4:CD8 ratio compared to the other study groups (Table 1).

**Figure 1:**
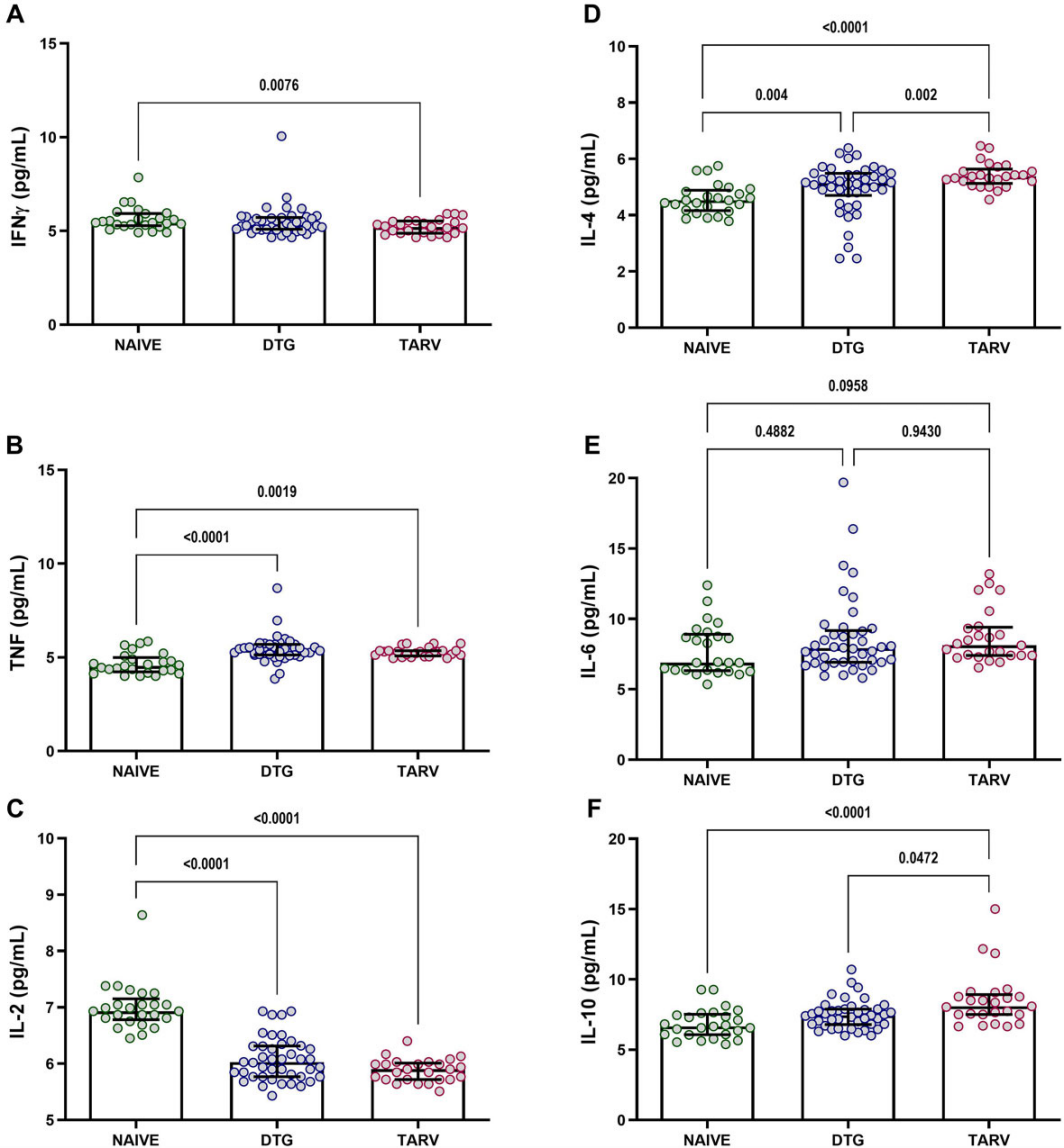
Comparison of the levels of cytokines interferon (IFN)-γ (**A**), tumor necrosis factor (TNF) (**B**), interleukin (IL)-2 (**C**), IL-4 (**D**), IL-6 (**E**), and IL-10 (**F**) between the dolutegravir (DTG) group, other antiretroviral treatment (TARV) group, and NAIVE group. Data are reported as median and interquartile range. P-values were derived from the Mann-Whitney U test.

To describe the study groups, the levels of inflammatory and anti-inflammatory cytokines were measured. Our findings showed that the DTG group had higher levels of TNF, IL-4, and IL-10 and lower levels of IL-2 than individuals in the NAIVE group. Furthermore, the TARV group had higher levels of TNF, IL-4, and IL-10 and lower levels of IFN-γ and IL-2 than the NAIVE group. Interestingly, the DTG group exhibited lower levels of IL-4 and IL-10 compared to the TARV group, indicating that PLWHA treated with another antiretroviral regimen had a more prominent anti-inflammatory profile (Figure 1).

In the correlation analyses, the DTG group showed positive correlations between the levels of CD4^+^ T lymphocytes and the levels of IL-6 and between the duration of treatment and the IL-10 levels. There was a negative correlation between CD4^+^ T lymphocyte levels and TNF levels, as well as between the duration of treatment and IL-2 levels. The NAIVE and TARV groups did not present any correlation in the present study (Supplementary Tables S1-S4). In addition, the correlation between the levels of cytokines in the study groups was performed, analyzing the balance between the inflammatory and anti-inflammatory response. The DTG group showed a significant positive correlation between the levels of IFN-γ and TNF and between IL-10 and IL-6, while the NAIVE group showed positive correlations between the levels of IL-10 and IFN-γ, IL-6, and IL-2, and between IFN-γ and IL-2. The TARV group did not show significant correlations between the analyzed cytokines.

## Discussion

Combined antiretroviral therapy has contributed significantly to improving the quality of life and consequently substantially increasing the survival of PLWHA, with a decrease in viral load and an increase in CD4^+^ T lymphocytes (3). The data presented in our study demonstrated this efficacy, regardless of the therapeutic regimen used, since both DTG and ART groups showed an increase in CD4^+^ T lymphocytes and an undetectable viral load, compared to the NAIVE group. In addition, we observed a decrease in CD8^+^ T lymphocytes in individuals on treatment, resulting in an increase in the CD4 ratio, with no statistically significant differences between the therapeutic regimens analyzed. The CD4 ratio is an indicator of great clinical relevance, since low values are associated with greater morbidity and mortality in PLWHA. Although DTG is a relatively recent addition to c-ART, our findings suggested that it also contributes to the recovery of this immunological marker. However, additional data are needed to fully assess the impact of this drug in three-drug regimens (9).

In addition to cell populations, drug combinations and/or drug classes that make up antiretroviral therapy may be associated with different immune responses and changes in the cytokine profile (10). Our findings showed that PLWHA treated with DTG have lower IL-4 and IL-10 levels than those treated with other types of c-ART. In terms of IL-4, our findings differed from the study by Szymańska et al. (8) who found lower levels of this cytokine in 30 HIV-infected males after one year of c-ART. However, when comparing individuals treated with PI and INI, they found no changes. Another study evaluated INI in drug combinations and observed a decrease in IL-4 six months after starting c-ART but did not find a statistical difference in the levels of this cytokine between the analyzed treatment regimen (11). We emphasize that most individuals in the study by Jianu et al. (11) were treated with raltegravir (INI).

IL-4 is a cytokine produced by CD4^+^ T lymphocytes, NK cells, basophils, and mast cells. It acts by stimulating B lymphocytes in producing IgG1 and IgE, increasing the expression of the major histocompatibility complex (MHC) class II in antigen presenting cells (APC), influencing the differentiation of macrophages, and inhibiting the secretion of pro-inflammatory cytokines (12). Therefore, we hypothesized that the decrease in IL-4 in the DTG group may be related to a more efficient modulation of the immune response, possibly with a balance between pro- and anti-inflammatory cytokines. This results in reduced viral replication and more effective control of the infection by decreasing the immunosuppressive influence of IL-4. Our findings, however, showed no difference in the levels of pro-inflammatory cytokines.

Although our study did not assess treatment adherence, a previous study suggests that high IL-4 levels are associated with lower adherence to antiretroviral treatment (13). This suggests that patients with high IL-4 levels, as in the TARV group, may have a suboptimal immune response due to poor treatment adherence, resulting in less effective infection control.

The DTG group showed significantly lower serum IL-10 levels compared to the TARV group, corroborating the study by Jianu et al. (11), who observed a significant decrease in plasma IL-10 levels in PLWHA under c-ART that included DTG. However, the authors emphasized that the result should be considered with caution due to the small sample size (n=4). Another previous study compared INI and PI in PLWHA under treatment for at least one year and observed lower levels of IL-10 in individuals using an INI, specifically elvitegravir (14). IL-10 is recognized as a crucial cytokine for immune homeostasis, regulating the intensity of the inflammatory response and preventing excessive tissue damage (15). In the context of HIV infection, elevated IL-10 levels are associated with chronic immune activation, often as a compensatory mechanism to mitigate inflammation resulting from viral replication.

Our study, which showed significantly lower levels of IL-10 in the DTG group compared to the TARV group, suggested that the TDF/3TC/DTG regimen may be more efficient in suppressing viral replication. This could be related to better control of infection in viral reservoirs, such as tissue macrophages, which would result in less chronic activation of the immune system and consequently lower IL-10 production (16). In addition, a previous study showed that regimens containing integrase inhibitors such as DTG can reduce inflammation and consequently immune activation compared to other classes of antiretrovirals and have a lower tendency to exacerbate the production of cytokines, which reinforces our data (17).

The reduction in IL-10 observed in the DTG group can be indicative of lower immune activation and possibly lower immune exhaustion, demonstrating that the TDF/3TC/DTG regimen is more effective in reducing chronic inflammation. This reduction helps delay immune exhaustion, a crucial factor since chronic immune activation is associated not only with HIV progression but also with premature aging and the development of diseases such as cardiovascular and neurodegenerative diseases (18).

On the other hand, even with lower levels of IL-10 in the DTG group, we observed a positive correlation between this cytokine and treatment duration. Initially, the DTG-containing regimen may suppress IL-10 production, favoring a more effective antiviral response. However, as the viral load decreases and the inflammatory state is controlled, the need to maintain immune homeostasis increases, leading to a gradual increase in IL-10. This increase may be related to the immune regulation necessary to deal with the persistence of viral reservoirs (19). Thus, the observed correlation suggests that, over time, IL-10 plays a role in modulating the residual immune response, contributing to the immune balance in patients treated with DTG.

Our study thus provides new data on the immunological effects of DTG, especially in the context of cytokines such as IL-4 and IL-10, for which there are still few consistent studies. The lower levels of IL-4 and IL-10 in the DTG group compared to ART reinforced the hypothesis that this antiretroviral regimen may be more efficient in reducing the chronic anti-inflammatory profile in PLWHA. This is of clinical relevance, as it suggests that DTG therapy may help control the inflammatory state compared to other treatments. In addition, the increase in CD4^+^ T lymphocytes and the reduction in CD8^+^ T lymphocytes in the treatment groups highlighted the efficacy of antiretroviral treatment in improving immune function.

Limitations of the study include the exclusion of patients with low CD4^+^ counts, which restricted sample selection and prevented the inclusion of individuals with the most severe form of infection (AIDS). This criterion limited the variability of the group analyzed, focusing on patients with better immunological status, which may affect the generalization of the results. In addition, possible confounding factors such as adherence to treatment and patient lifestyle were not assessed, which may have influenced cytokine levels and consequently the study findings.

Furthermore, our study opted to use the CBA system, which enables simultaneous measurement of multiple analytes in biological sample volumes that are too small. A similar methodology would be the Luminex system. The accuracy and reproducibility of cytokine measurement by these methods is highly dependent on the availability of high-quality standard curves. The variability within a kit and between kits was high for the standard curves generated with the CBA kits. In contrast, standard curves generated using Luminex technology had low variability within a kit and between kits (20), being more reproducible. An advantage of the CBA over the Luminex system is the possibility of using any flow cytometer model that has the Beckton Dickson software, which is a reality in many research laboratories, instead of using specialized detection equipment.

## Supplementary Material

Click here to view [pdf].
