# Supplementary material for: Evaluation of cytokine levels in HIV-infected individuals on therapy with tenofovir, lamivudine, and dolutegravir
Source: Braz J Med Biol Res. 2025 May 9;58:e14442. doi: 10.1590/1414-431X2025e14442 (PMC12068765; doi:10.1590/1414-431X2025e14442)
Supplement: Supplementary file 1 [file 1414-431X-bjmbr-58-e14442-suppl.pdf]

Table S1. Association between $CD4^+$ T cells and cytokines.

$CD4^+$ T cells counts	IFN-γ	TNF	IL-2	IL-4	IL-6	IL-10
NAIVE						
Spearman r	–0.17	–0.02	0.03	–0.11	0.10	–0.18
P	0.40	0.92	0.87	0.59	0.62	0.38
DTG						
Spearman r	–0.19	–0.35	–0.20	0.25	0.33	0.26
P	0.21	**0.01**	0.18	0.10	**0.03**	0.09
TARV						
Spearman r	0.25	–0.02	–0.13	–0.06	–0.14	0.13
P	0.21	0.91	0.52	0.75	0.48	0.51

Spearman correlation. Values in bold type are statistically significant (P<0.05). DTG: dolutegravir; TARV: other antiretroviral therapy; IFN-γ: interferon gamma; TNF: tumor necrosis factor; IL: interleukin.

Table S2. Association between CD8+ T cells and cytokines.

$CD4^+$ T cell counts *versus*	IFN-γ	TNF	IL-2	IL-4	IL-6	IL-10
NAIVE						
Spearman r	0.01	0.13	0.11	0.03	–0.23	0.003
P	0.95	0.50	0.58	0.87	0.26	0.98
DTG						
Spearman r	–0.11	–0.16	–0.24	0.12	0.06	–0.04
P	0.48	0.28	0.11	0.42	0.69	0.75
TARV						
Spearman r	0.34	0.07	–0.25	0.28	–0.007	0.17
P	0.09	0.73	0.21	0.17	0.97	0.41

Spearman correlation. DTG: dolutegravir; TARV: other antiretroviral therapy; IFN-γ: interferon gamma; TNF: tumor necrosis factor; IL: interleukin.

Table S3. Association between viral load and cytokines.

Viral load counts *versus*	IFN-γ	TNF	IL-2	IL-4	IL-6	IL-10
NAIVE						
Spearman r	0.006	–0.27	–0.009	–0.28	0.15	0.42
P	0.97	0.24	0.96	0.21	0.50	0.06

Spearman correlation. IFN-γ: interferon gamma; TNF: tumor necrosis factor; IL: interleukin.

Table S4. Association between treatment duration and cytokines.

Duration of treatment	IFN-γ	TNF	IL-2	IL-4	IL-6	IL-10
DTG						
Spearman r	0.02	–0.18	–0.43	0.25	0.09	0.42
P	0.86	0.24	0.003	0.09	0.54	0.004
TARV						
Spearman r	–0.15	0.21	0.11	0.06	0.01	0.05
P	0.47	0.29	0.58	0.74	0.94	0.77

Spearman correlation. DTG: dolutegravir; TARV: other antiretroviral therapy; IFN-γ: interferon gamma; TNF: tumor necrosis factor; IL: interleukin.
